# “Hybrid” lesion of desmoplastic and conventional ameloblastoma: immunohistochemical aspects

**DOI:** 10.1016/S1808-8694(15)31030-2

**Published:** 2015-10-19

**Authors:** Jean Nunes dos Santos, Veronica Ferreira De Souza, Roberto Almeida Azevêdo, Viviane Almeida Sarmento, Lélia Batista Souza

**Affiliations:** a PhD in oral pathology, Associate Professor at the School of Dentistry of the Federal University of Bahia.; b MSc in Dentistry, Assistant Professor of Dentistry - Southeast State University - Bahia.; c PhD in Dentistry, Associate Professor at the School of Dentistry of the Federal University of Bahia; d PhD in stomatology, Associate Professor at the School of Dentistry of the Feira de Santana State University.; e PhD in oral pathology, Associate Professor at the School of Dentistry of the Federal University of the Rio Grande do Norte. Federal University of Bahia. We thank FAPESB (partnership 032299, 200/04) for financial support.

**Keywords:** ameloblastoma, extracellular matrix

## Abstract

Ameloblastoma is a benign epithelial odontogenic tumor and is the most commonly encountered odontogenic tumor in the jaws. Histologically, ameloblastomas occur in different patterns, including plexiform pattern and follicular pattern. “Hybrid “ lesion of ameloblastoma is a tumor variant in which histologically, areas of follicular or plexiform ameloblastoma coexist with characteristic areas of ameloblastoma exhibiting pronounced stromal desmoplasia (desmoplastic ameloblastoma). The purpose of this article is to present a case of “hybrid” lesion of desmoplastic ameloblastoma (AD) and conventional, and investigate extracellular matrix proteins such as tenascin, fibronectin, and type I collagen.

## INTRODUCTION

Ameloblastomas are a common benign neoplasia of maxillary bones, originated from the remains of the dental plate. Histologically, it occurs in many patterns, including the types: follicular, plexiform, acanthomatous, of granular cells, of basal cells and the desmoplastic. The latter was initially described by Eversole et al.^1^, in 1984. It is usually characterized by a pronounced stroma collagenization, which is permeated by small islets and cords of the tumoral odontogenic epithelium. It also bears an uncommon radiographic aspect and a marked difference in anatomical location when compared to the conventional ameloblastoma.

In recent years, the literature has described “hybrid lesions” of desmoplastic ameloblastoma and conventional ameloblastoma, that are microscopically characterized by presenting areas of follicular or plexiform ameloblastoma coexisting with areas of desmoplastic ameloblastoma^2^, ^3^.

Moreover, many studies have shown the importance of the extracellular matrix (ECM) in the modulation of neoplastic cell behavior and in the histomorphologic aspects of tumoral cells^4^. Thus, the present study aims at reporting a case of a “hybrid” desmoplastic and conventional ameloblastoma (AB) in the mandible, highlighting the immune-histochemical findings related to the expression of tenascin and fibronectin proteins and type I collagen in the stroma of the lesion.

## CASE REPORT

A 36 year old man came for dental care complaining of a painless volume increase within his mouth. Extra-oral clinical exam showed a slight volume increase on the right side of the mandible. Inside the mouth we found a hard volume increase going from the inferior cusp all the way to the pre-molar region covered by normal-looking mucosa. The dental units involved were somewhat shifted, with slight mobility and without pulp involvement. Radiographic exam revealed a radiolucent area of ill-defined outline in the region between units 33 and 37. Under clinical suspicion of ameloblastoma, we carried out an incisional biopsy of which the histopathological diagnosis was confirmed. The lesion was then surgically removed by block resection, preserving the lower border of the mandible. The subsequent histopathology exam established the diagnosis of a hybrid desmoplastic and follicular ameloblastoma lesion.

For the immunehistochemical technique we prepared 3µm sections and submitted those to the streptavidine biotin method. The antibodies used in this study, their sources, dilutions and protocols are listed on Table 1. The cross sections were submitted to antigenic recovery, using 1% pepsin (1.0 g of pepsin in 100ml of 10% chloridric acid solution in pH of 1.8 for 2 hours at 37oC) and steam heat. We also included proper positive and negative controls. Diaminobenzidine was used as a chromogen and the cross sections were counter dyed by Mayer's hematoxylin.

### Histopathology findings

**Figure 1:**
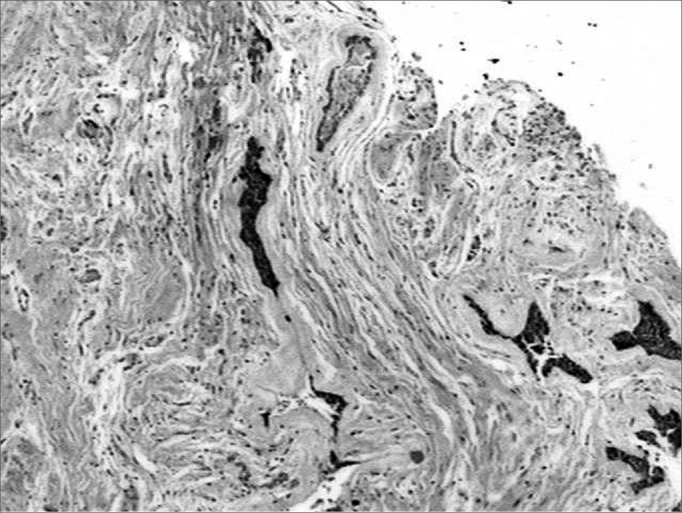
Connective stroma densely collagenized and permeated by compressed epithelial neoplastic islets, as well as by epithelial islet with peripheral cells arranged in palisade (H/E, 40x).

The histopathology exam showed areas of desmoplastic and follicular ameloblastoma. In the former we observed tumoral epithelial islets spread in a densely collagenized connective stroma (Figure 1). These tumoral islets, which seemed to be compressed, were made up of cuboid or spindle-shaped cells showing cellular periphery in palisade absent. The second histology subtype was represented by epithelial islets of which peripheral cells were distributed in palisade, similar to the ameloblastomas of the enamel organ (Figure 1). The cells in the central region were similar to the stellar reticulum of the enamel organ. Often times these cell arrangements showed cystic degeneration and, eventually, squamous metaplasia. The stroma was less dense and made up of scarce lymphocytes.

### Immune-histochemical findings

 

### Tenascin

**Figure 2:**
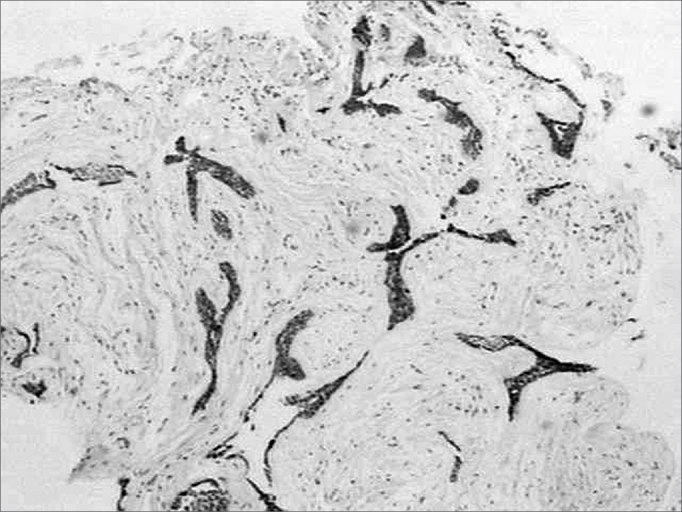
Lack of immune marking for tenascin in the lesion stroma (streptavidine biotin, 40x).

The tenascin marking pattern was of the fibrillar type, with intensity varying from mild to moderate in the stroma corresponding to the follicular ameloblastoma. In the stroma corresponding to the DA we did not observe markings for this immune protein in the ECM (Figure 2). In the follicular ameloblastoma tumoral islets, which showed cystic degeneration, we saw one intense and homogenous immune marking, sometimes linear, outlining the cystic spaces. Sometimes these tumoral islets that showed squamous metaplasia were also immune marked.

### Fibronectin

**Figure 3:**
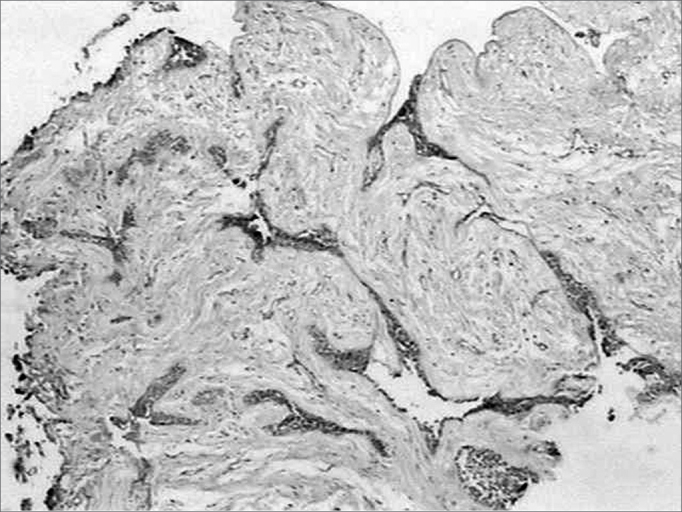
Fibrillar fibronectin pattern in the tumor stroma (streptavidine biotin, 40x).

All along the tumoral stroma, of the follicular ameloblastoma or the desmoplastic, there was a strong immune marking distributed in a fibrillar pattern (Figure 3). On the epithelium/mesenchyma interface we noticed a strong linear marking, specially in the areas that corresponded to the conventional ameloblastoma. Some epithelial cells in the tumoral islets under cystic degeneration also expressed this protein.

### Collagen I

**Figure 4:**
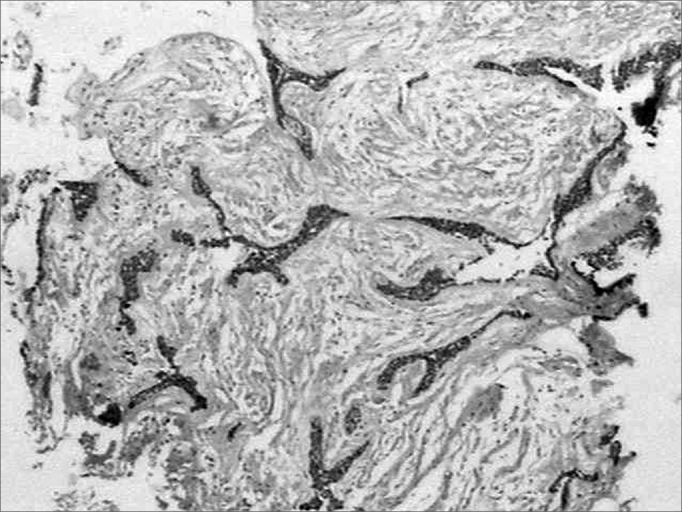
Strong affinity with type I collagen in a fibrillar pattern on the desmoplastic stroma of the lesion (streptavidine biotin, 40x).

Similar to fibronectin, all along the tumoral stroma of the hybrid lesion in the desmoplastic ameloblastoma, there was an intense immune marking for this ECM protein, in a fibrillar pattern involving the tumoral islets (Figure 4). Sometimes, on the epithelium/mesenchyma interface, we noticed intense linear immune marking, specially on the areas corresponding to the conventional ameloblastoma. Moreover, we noticed lack of type I collagen inside these tumoral cells.

## DISCUSSION

In the literature we noticed that the DA represented the odontogenic tumor of the least incidence. Among 89 cases of ameloblastomas studied by Takata et al.^5^, seven (7.9%) were diagnosed as DA; and only (1.1%) as “hybrid lesion”. In Japan, the DA was responsible for 5.3% of all the cases of intraosseus ameloblastomas diagnosed in 27 years^6^. The clinical aspects reported in the present case match those mentioned in the literature, where the DA is presented as a painless volume increase on the face and with a slight predilection for males^5^, ^7^.

Radiographically, the DA presents aspects which are very different from those of the conventional ameloblastoma, characterizing itself as a radiolucent lesion of unclear limits, resembling soap bubbles^7^. In many cases, the lesion appears as a radiolucent area with radiopacity points that mimic fibro-osseous lesions^8^.

Since it presents its own clinical and radiographic characteristics, the desmoplastic ameloblastoma (DA) has been considered by some authors as a clinical and pathological distinct entity^2^, ^5^. This was recently confirmed by the World Health Organization (WHO), of which classification organized the ameloblastomas in solid, extraosseous, desmoplastic and unicystic^9^.

The histopathological characteristics described for the present case are in agreement with the diagnostic criteria established for a “hybrid lesion” of desmoplastic and conventional ameloblastomas according to Waldron and El-mofty^2^. Thus, such lesion is characterized as containing DA areas in association with typical areas of follicular ameloblastoma. The areas corresponding to the desmoplastic variant is characterized by small nests and cords of odontogenic tumoral epithelium within an abundant stroma densely collagenized, and this makes those tumoral islets to seem compressed. The peripheral cells lose that characteristic that is so similar to ameloblasts with polarity inversion. Moreover, the central portion of these tumoral islets may present hypercellularity, with occasional squamous metaplasia and central keratinization foci^8^. In the latter, we must include the differential diagnosis of odontogenic squamous tumor^9^. Other tumors such as the odontogenic fibroma must also be considered in the DA differential diagnosis^7^.

In the present case we did not observe an exuberant inflammatory reaction, although one of the alternatives used to justify the desmoplasia would be the phenomena accruing from inflammation.

We know that the most important characteristic of a DA is the extensive collagenization present in the lesion stroma, also called desmoplasia^1^, ^2^, ^5^, ^10^. It has been proposed that such phenomenon originates from a new protein synthesis in the extracellular matrix^11^. Such components play a fundamental role in the support, adhesion, migration and differentiation of tumoral cells, interfering on the behavior and modulation of these cells^12^, ^13^. Thus, we investigated the distribution of tenascin, fibronectin and type I collagen in the case hereby reported.

Tenascin is a protein from the extracellular matrix with a very restrict distribution pattern. It is expressed during embryogenesis, specially in the areas of epithelium-mesenchyma interaction^14^. In cases of ameloblastoma and adenomatoid odontogenic tumors, tenascin is mainly located on the parenchyma-stroma interface, suggesting an important participation of this protein in the relationship between the tumoral cells and the adjacent connective tissue^12^, ^13^. In the case hereby reported, having tenascin only in the areas corresponding to the follicular ameloblastoma could explain the more aggressive behavior of this histologic pattern, since tenascin has been observed in unstable environments such as the ones created by neoplasias^4^. The tumoral islets are also positive for tenascin, specially in those areas that show some cystic degeneration. Such finding was also reported by Mori et al.^12^ and Medeiros^13^, who related the immune-location of this protein to the mechanisms that form and expand cystic spaces.

Fibronectin represents an adhesion protein of high molecular weight, of which distribution follows that of lose connective tissue. Its presence has been associated to cellular functions such as adhesion, migration, proliferation and differentiation, being related to important moments of embryogenesis, tissue development and healing. During odontogenesis, it interferes on the maturation of odontoblasts^14^.

In ameloblastomas, fibronectin is produced by the cells present in the tumoral stroma^14^. Its distribution is relatively uniform in the stroma of many histologic types of ameloblastoma. Its distribution is relatively uniform in the stroma of various types of ameloblastoma, with greater intensity in the epithelium-mesenchyma interface, except in the desmoplastic variant, probably due to the lack of similarities with the pre-ameloblasts on the peripheral epithelial cells^13^. A similar aspect was also shown in the present case, where fibronectin was strongly marked on the epithelium-mesenchyma interface of the tumoral areas corresponding to the follicular ameloblastoma.

Tenascin has been described as capable to modulate the action of other molecules in the extracellular matrix, affecting cell shape and blocking fibronectin^4^. Such aspect was not observed in our study, having in mind that fibronectin was seen in the areas also marked by tenascin.

Type I collagen is the major structure present in skin, tendon and bones, and it is also the main organic compound present in dentin^15^. In desmoplastic ameloblastoma, there is an intense immunepositiveness of this protein when compared to the other histologic subtypes^13^. In a similar way, type I collagen presented strong immune marking in the stroma of the present case, reflecting the high grade of this protein present in DAs.

## FINAL COMMENTS

Although tenascin, fibronectin and type I collagen participate on the tumoral modulation of the “hybrid lesion” follicular and desmoplastic ameloblastoma, the biologic mechanism of desmoplasia calls to newer investigations regarding the other extracellular matrix components, as well as other study methods, in an attempt to understand the conspicuous stromal collagenization and its relationship with the epithelial component of the lesion.

This article has received corrections in agreement with the ERRATUM published in Volume 72 Number 6.
